# Association of a BMP9 Haplotype with Ossification of the Posterior Longitudinal Ligament (OPLL) in a Chinese Population

**DOI:** 10.1371/journal.pone.0040587

**Published:** 2012-07-19

**Authors:** Yuan Ren, Zhi-zhong Liu, Jie Feng, Hong Wan, Jun-hua Li, Hao Wang, Xin Lin

**Affiliations:** 1 Department of Orthopaedics, Beijing Tiantan Hospital, Capital Medical University, Chongwen District, Beijing, China; 2 Department of Clinic Laboratory, Beijing Tiantan Hospital, Capital Medical University, Chongwen District, Beijing, China; 3 Department of Institute of Neurosurgery, Beijing Tiantan Hospital, Capital Medical University, Chongwen District, Beijing, China; Central China Normal University, China

## Abstract

Direct or *ex vivo* BMP9 adenoviral gene therapy can induce massive bone formation at the injection sites and clearly promote spinal fusion. A comprehensive analysis of the osteogenic activity indicated that BMP9 was one of the most potent inducers of osteogenic differentiation both in vitro and in vivo among 14 types of human BMPs. However, genetic variations and whether they correlated with OPLL were not considered. We have sequenced the complete BMP9 gene in 450 patients with OPLL and in 550 matched controls. Analyses were performed on single markers and haplotypes. Single marker tests identified 6 SNPs, among which the minor alleles of rs7923671 (T>C; *P = 0.0026*; OR: 1.33, CI: 1.10–1.60), rs75024165 (C>T, Thr304Met; *P<0.001*; OR: 1.76, CI: 1.47–2.12) and rs34379100 (A>C; *P<0.001*; OR: 1.52, CI: 1.27–1.82) were associated with OPLL. Logistic regression analysis showed that the additive model of rs75024165 (TT vs. CT vs. CC; *P<0.001*; OR: 1.74) and rs34379100 (CC vs. AC vs. AA; *P = 0.003*; OR: 1.95) retained statistical significance when adjusted for clinical and demographic characteristics. Linkage disequilibrium (LD) analysis identified one 3 kb block of intense LD in BMP9 and one specific haplotype, CTCA (*P<0.001*; OR: 2.37), that contained the OPLL-associated risk alleles and was a risk factor for OPLL. This haplotype is associated with increased severity of OPLL, as shown by the distribution of ossified vertebrae in patients with OPLL (*P = 0.001*). In summary, in the Chinese population studied, SNPs in the BMP9 gene appear to contribute to the risk of OPLL in association with certain clinical and demographic characteristics. The severity of OPLL seems to be mediated predominantly by genetic variations in a 3kb BMP9 locus with the specific haplotype CTCA.

## Introduction

Ossification of the posterior longitudinal ligament (OPLL) is characterized by ectopic new bone formation replacement of ligamentous tissues of the spine and often leads to various degrees of myeloradiculopathy as a result of compression of the spinal cord and nerve roots [1], [2]. OPLL of the spine has been predominantly reported for Asian populations with a prevalence of 1.9%–4.3% in Japanese (aged >30 years) and 0.44%–8.92% in Chinese [3], [4], [5], [6], [7]. The etiology of OPLL is not clear up to date, although high body mass index (BMI), high peripheral bone mineral density (BMD), dietary habits, aging and glucose intolerance, have been proposed causes of OPLL [8], [9], [10], [11], [12]. On the basis of nationwide pedigree surveys, and surveys of twins and analysis of human leukocyte antigen haplotypes, genetic factors have also been suggested to be involved in the development of OPLL [13], [14], [15], [16]. Previous studies have shown that single nucleotide polymorphisms (SNPs) in the transforming growth factor-β, nucleotide pyrophosphatase (NPPS), leptin receptor, collagen 6A1(COL6A1), bone morphogenetic protein 2 (BMP2) and bone morphogenetic protein 4 (BMP4) as well as estrogen receptor (ER) and interleukin-1 (IL-1) genes are associated with the development of OPLL [17], [18], [19], [20], [21]. However, only BMPs were shown to induce ectopic ossification when these proteins are implanted subcutaneously [22]. Our research group has previously shown that BMP2 and BMP4 synonymous and non-synonymous SNPs are associated with the occurrence and severity of OPLL in a small Chinese population, although little evaluation of the clinical and demographic backgrounds of the participants was carried out [18], [36]. In this study we focused on the role of Bone morphogenetic protein 9 (BMP9), also known as growth and differentiation factor–2 (GDF-2) on the development of OPLL. BMP9 was originally identified in fetal mouse liver cDNA libraries, but it is a relatively uncharacterized member of the BMP family [23]. It has been reported that BMP9 may play a role in regulating glucose and iron homeostasis in liver [24], [25]. Interestingly, a comprehensive analysis of the osteogenic activities of the 14 types of human BMPs demonstrated that BMP9 was one of the most potent inducers of osteogenic differentiation both *in vitro* and *in vivo* [26], [27]. BMP9 was also shown to be a potent modulator of cartilage development *in vitro* and may play a role in gene therapy for bony defects, craniofacial bone repair, and nonunion bone fracture repair [28], [29], [30]. Additional studies have demonstrated that human mesenchymal stem cells (hMSCs) transfected with hBMP9 could lead to ectopic bone formation in rabbit and induce spinal fusion in rodents [31], [32], [33]. Most recently, Leblanc *et al.* found that injecting BMP9 protein into damaged muscle can directly induce muscle heterotopic ossification [34]. Up to know only one study of BMP9 response to novel activin receptor-like kinase 1 (ALK1) mutants derived from non synonymous SNPs in hereditary hemorrhagic telangiectasia type 2 (HHT2) patients has been reported [35] and no previous studies have assessed BMP9 variations. In the current study we hypothesized that BMP9 might be involved in OPLL, and we evaluated the overall polymorphisms of the BMP9 gene in association with certain clinical and demographic characteristics in a large group of Chinese patients with OPLL.

## Materials and Methods

### Participants

A case group that consisted of 450 Chinese patients with the common disease type of cervical OPLL and a control group of 550 healthy participants without cervical OPLL were enrolled in this study. All participants were recruited from Grade III, class A hospitals in mainland China. The diagnosis of OPLL was based on imaging findings, which included radiographs, computed tomography (CT) scans, and magnetic resonance imaging (MRI) of the cervical spine, in accordance with the criteria reported by Tsuyama [37]. The severity of OPLL was determined by the number of ossified cervical vertebrae observed using lateral radiographs and CT images. Patients with ankylosing spondylitis and metabolic diseases associated with OPLL, such as hypophosphatemic rickets/osteomalacia, osteoporosis, diffuse idiopathic skeletal hyperostosis (DISH), and hyperparathyroidism, were excluded from the study on the basis of radiographic and biochemical examinations. Participants who had taken drugs such as estrogen, progesterone, glucocorticoids, bisphosphonates, alfacalcidol, and calcitriol were also excluded.

Blood samples were collected from all participants. The study protocol was approved by the ethical committee of Beijing Tiantan Hospital, Capital Medical University with the following reference number: 200824 and written informed consent was obtained from all participants before the study. To evaluate the potential risk factors of OPLL, a standardized questionnaire [11] was used and related anthropometric and lifestyle determination methods are shown in Material S1.

### Genotyping of BMP9

Genomic DNA was extracted from peripheral blood leukocytes using a Wizard Genomic DNA Purification Kit (Promega, Madison, WI, USA). The BMP9 gene was amplified by the polymerase chain reaction (PCR) with a standard protocol [38] in a total of 5 overlapping fragments and sequenced. The reactions were performed. Different thermocycling parameters were used for different primer pairs (Table S1). Sequencing reactions were performed using the Big Dye Terminator v3.1 Cycle Sequencing Kit (Applied Biosystems, Foster City, CA, USA ), and the extension products were analyzed on an ABI 3730XL POP7 DNA sequencing analysis 5.2 system (Applied Biosystems). All described data about polymorphisms were discovered in our study, but before our experiments, all these rs-ID (rs3758496, rs12252199, rs7923671, rs75024165, rs34379100, rs9421799) have also been reported in GeneBank.

### Statistical Analysis

Hardy–Weinberg equilibrium and the genotypic and allelic distributions were evaluated using χ2 tests. The statistical power was calculated using Quanto v1.2.3 software. To control for potential confounding variables, we constructed a logistic regression model with the occurrence of OPLL as an outcome variable. The predictor variables included age, gender, sleeping habits, BMI, FBG, BMD, exercise, smoking, alcohol consumption, and the additive model for each SNP in BMP9. These clinical and demographic factors, which have been associated with the occurrence of OPLL in previous studies of other Asiatic populations, were re-evaluated in our study [8], [9], [10], [11], [12]. A backwards stepwise regression analysis (binary logistic regression) was performed to assess the independent effects of these covariates in the logistic regression model for OPLL and estimate the odds ratios (ORs) and 95% confidence intervals (95% CIs) for the two groups with regard to the risk of developing OPLL. A nonparametric test (Mann–Whitney exact test) was employed to compare the number of ossified cervical vertebrae between the two groups. The data are expressed as the means ± SD. SPSS 13 software (SPSS, Chicago, IL, USA) was used to analyze the data and calculate P values, odds ratios, and 95% CIs. Haplotype-based association studies were performed using both the Haploview V.3.32 and the SHESIS software platforms. Unless indicated otherwise, a P value less than 0.05 was considered statistically significant.

## Results

### Allele and Genotype Findings

The clinical and demographic characteristics of OPLL patients and the control group are shown in Table 1; the two groups did not differ significantly in each item, implying that they are comparable. The BMP9 genes of all participants were sequenced, and 6 SNPs were identified and genotyped (minor allele frequency in cases >0.05). The characteristics of the SNPs are given in Table S2. Allele and genotype frequencies of all SNPs were in Hardy–Weinberg equilibrium in both groups. Single-marker tests, which were based on an allelic model, revealed associations between an increased risk of OPLL and the minor alleles of rs7923671 (T>C; *P = 0.0026*; OR: 1.33,CI: 1.10–1.60), rs75024165 (C>T, Thr304Met; *P<0.001*; OR: 1.76, CI: 1.47–2.12), and rs34379100 (A>C; *P<0.001*; OR: 1.52,CI: 1.27–1.82).

**Table 1:** Clinical and demographic characteristics.

VARIABLES	OPLL N = 450	Control N = 550
Gender(male/female)	216/234	230/320
Age (years) ≥60, 30–60, <30	50.21±17.54 Range(20–90) 113/286/51	50.70±18.11 Range(19–90) 63/345/142
Regular sleeping habit	130 (28.89)	164 (29.82)
BMD T≥1 high, −1<T<1 Normal, T<−1 low	0.07±1.65 Range(−2.2–4) 107/207/136	−0.39±1.91 Range(−4.01–3.98) 90/266/194
BMI(kg/m2) ≥25, 18.5–25, <18.5	26.07±3.45 Range(18.51–31) 315/135/0	25.92±3.38 Range(18.53–30.49) 368/182/0
FBG(mmol/l) ≥7.0, <7.0	6.53±2.15 Range(3.9–12.1) 116/334	5.66±1.39 Range(3.9–11.7) 37/513
Exercise (<Once a week)	141(31.33)	164(29.82)
Smoking(yes)	289(64.22)	350(63.64)
Alcohol drinking (≥Once a week)	373(82.89)	453(82.36)

**Note**: The data represent the means±SD. BMI: body mass index, FBG: fasting blood glucose level, BMD: Bone Mineral Density.

Table S2 and Table S3 show that the power of the minor allele frequencies was more than 90% for all SNPs with *P<0.001*. The power of the ossified vertebrae averages was more than 90% for related clinical and demographic characteristics with *P<0.05* (except regular sleeping habit: 79.5%, normal exercise: 79.5%, and smoker: 87.9%).

The backward regression analysis, which was based on an additive model and adjusted for clinical and demographic characteristics, showed significant associations between the occurrence of OPLL and the additive model of rs75024165 (TT vs. CT vs. CC; *P<0.001*; OR: 1.82,CI: 1.42–2.32) and rs34379100 (CC vs. AC vs. AA; *P = 0.003*; OR:1.95,CI:1.15–1.96). Gender (male vs. female; *P = 0.043*; OR: 1.32, CI: 1.01–1.73) and FBG (≥7.0 vs. <7.0, mmol/L; *P<0.001*; OR: 5.42, CI: 3.60–8.15) showed statistically significant differences between the case and control groups (Table 2).

**Table 2:** Analysis of the genotype distribution in BMP9 between the OPLL and control groups.

SNP- No.	rs-ID	Number/Frequency (AA/AB/BB)		P(χ^2^)	P(logistic)	Score	Wald	OddsRatio	95% CI
		(OPLL)	(controls)						
1	rs3758496	115(0.26)/212(0.47)/123(0.27)	156(0.28)/263(0.48)/131(0.24)	0.3772	0.301	1.07		–	–
2	rs12252199	216(0.48)/200(0.44)/34(0.08)	272(0.49)/223(0.41)/55(0.10)	0.2647	0.233	1.42		–	–
3	rs7923671	179(0.40)/196(0.44)/75(0.17)	247(0.45)/254(0.46)/49(0.09)	0.0009	0.163	1.95		–	–
4	rs75024165	128(0.28)/234(0.52)/88(0.20)	258(0.47)/230(0.42)/62(0.11)	<0.001**	<0.001**		23.17	1.82	1.42–2.32
5	rs34379100	150(0.33)/222(0.49)/78(0.17)	255(0.46)/235(0.43)/60(0.11)	<0.001**	0.003		8.98	1.95	1.15–1.96
6	rs9421799	102(0.23)/232(0.52)/116(0.26)	138(0.25)/273(0.50)/139(0.25)	0.6665	0.004		8.52	0.69	0.53–0.88

**Note:** P (χ^2^) calculated by χ^2^ test of the additive model. P (Logistic) indicates correction for clinical and demographic characteristics and the additive model of each SNP using the binary logistic method (backward). **Gender (male)  =  (WALD: 4.10;** ***P*** **=** ***0.043*** **; OR: 1.32, CI: 1.01–1.73), FBG =  (SCORE: 65.86;** ***P<0.001*** **; OR: 5.42, CI: 3.60–8.15), rs7923671, rs75024165 and rs34379100 show statistical significance** ***(P<0.05).***

* = *P* ***<*** *0.05*,

** = *P* ***<*** *0.01*,

*** = *P* ***<*** *0.001.*

### Association between BMP9 Haplotypes and Occurrence of OPLL

**Figure 1:**
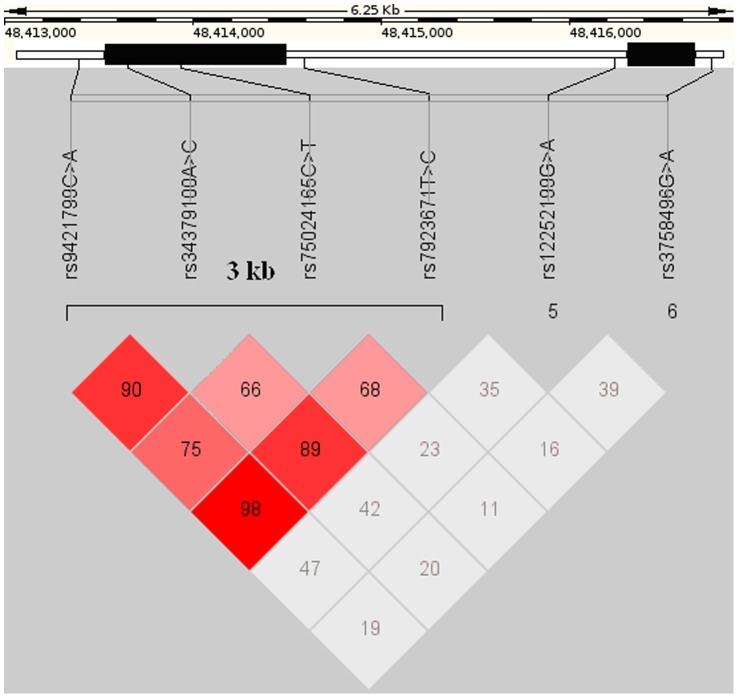
Genomic organization and linkage disequilibrium (LD) mapping of SNPs in a 6 kb genomic region within the BMP9 gene. The left black bar indicates the exon 2 and the right black bar indicates the exon 1 coding area. Each box represents the D’ value of the LD (range from 0 to 1) between pairs of SNPs. Red, strong LD; grey, high D’/low LOD value; white, low D’.

We identified one 3 kb block of intense LD. This block encompassed SNPs rs9421799–rs7923671, and the linkage equilibrium coefficient D’ reduced gradually from the middle of the block toward rs9421799 and rs7923671 at the boundaries (Fig. 1). In this block, we identified several common single SNP haplotypes that covered together more than 60% of the individuals within both groups. Analysis of haplotype-based associations between the case and control groups found that the haplotype CTCA, which included the risk (*) alleles for two SNPs (rs7923671 C, rs75024165 *T, rs34379100 *C, and rs9421799 A) was associated strongly with OPLL (*P<0.01*; OR: 2.37, CI: 1.93–2.90) (Table 3).

**Table 3:** Haplotypes of the *BMP9* SNPs in cases and controls.

Haplotypes	rs7923671T>C	rs75024165C>T	rs34379100A>C	rs9421799C>A	Frequency		χ2(df = 1)	P(χ2)	P(MonteCarlo)	OR	95%CI
					OPLL	Control					
Haplotype 1	C	T	C	A	0.37	0.19	70.22	<0.001	<0.01	2.37	1.93–2.90
Haplotype 2	T	C	A	C	0.43	0.44	1.73	0.1882	1.00	0.89	0.74–1.06
Haplotype 3	T	C	A	A	0.07	0.11	12.13	0.0005	1.00	0.58	0.42–0.79
Haplotype 4	T	T	A	A	0.04	0.04	<0.001	0.9988	1.00	1.00	0.64–1.56
Other haplotypes	–				0.09	0.22	–				

Note: Haplotype 1 includes the risk alleles of rs75024165 *T and rs34379100 *C (*P<0.01*).

* p-value of haplotype by χ^2^ and Monte Carlo (Number of permutation = 100).

### Association between BMP9 Haplotypes and Extent of OPLL Related to Clinical and Demographic Characteristics

**Figure 2:**
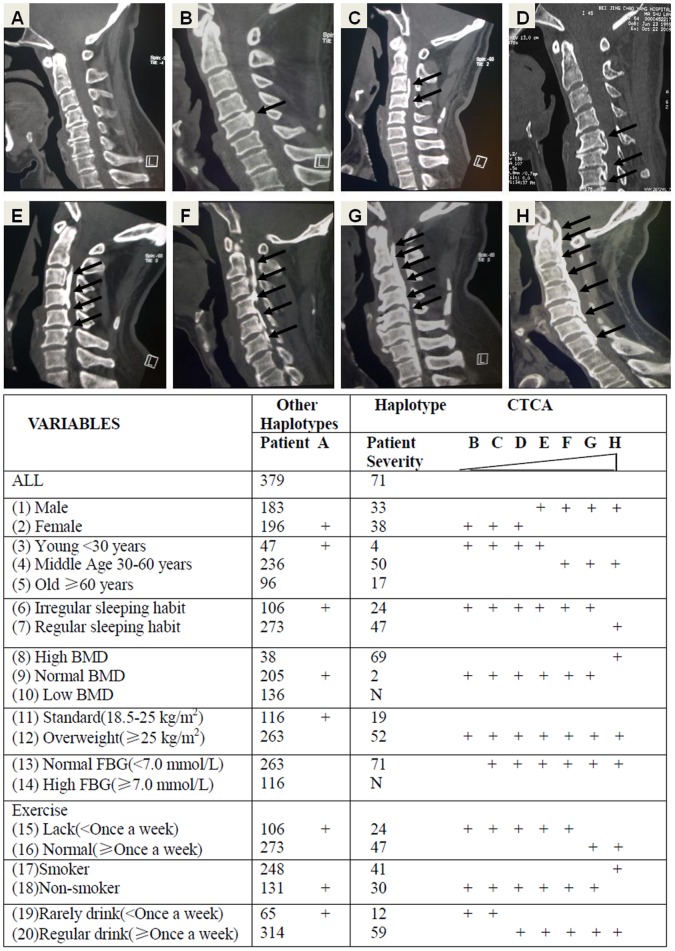
Association between BMP9 Haplotypes and Extent of OPLL related to clinical and demographic characteristics: The upper panel shows Two-dimensional computed tomography (2D-CT) pictures of OPLL severities in cervical spines (C2–7). Patients A: other haplotypes, Patients B–H: haplotype CTCA. The Table shows ossification severity (B–H) in the CTCA and other haplotype groups related to demographic and clinical characteristics.

The number of ossified vertebrae was analyzed in relation to the haplotype distribution in patients with OPLL who had been stratified by clinical and demographic characteristics. The results are shown in Fig. 2 and Table S3. Figure 2 shows two-dimensional computed tomography (2D-CT) scan pictures of OPLL patients with others (A) and the CTCA haplotype (B–H). As visible the degrees of ossifications in the CTCA haplotype group (B–H) is varying and the lower panel shows a scheme of the occurrence of ossification severities in the haplotype groups related to demographic and clinical characteristics. Patients with haplotype CTCA (71/450) developed a significantly (*P = 0.001*) greater number of ossified cervical vertebrae (3.41±1.58) than those who did not carry this haplotype (2.76±1.44). This effect was more obvious in patients with certain characteristics. The characteristics of smoker (*P = 0.003*), normal exercise (*P = 0.007*) and regular sleeping habit (*P = 0.007*) were associated with increased severity of OPLL in patients with the CTCA haplotype. In addition, normal FBG (*P<0.001*), middle age (*P<0.001*), regular drink (*P = 0.002*), high BMD (*P<0.001*), and being over weighted (*P = 0.002*) increased the extent of OPLL in patients with the CTCA haplotype significantly. The characteristics of male sex and high FBG might affect the initiation of OPLL significantly, in accordance with the findings of the logistic regression analysis, but only male sex (*P = 0.001*) was associated with an increase in the extent of heterotopic ossification in patients with the CTCA haplotype. Patients with the CTCA haplotype may suffer OPLL more severely as other haplotype patients, because they develop multiple cervical ossifications, but the severity is also related to other clinical and demographic characteristics. In addition, high FBG or low BMD seem to protect even CTCA haplotype patients against ossification. In summary, the CTCA haplotype is a major factor for ossification, but other factors as shown in Fig. 2 and Table S3 are contributors to the ossification severity.

## Discussion

To confirm that the BMP9 locus is associated with the occurrence of OPLL, we sequenced the complete 6 kb BMP9 genomic region and identified 6 SNPs. A LD test showed one block of intense LD, which encompassed SNPs rs9421799–rs7923671 (Fig. 1). Haplotype block analysis identified the haplotype CTCA as a risk factor for OPLL; this haplotype included the risk alleles of rs75024165 (T) and rs34379100(C) (Table 3). Finally, the results of our study suggested that the CTCA haplotype was associated with increased severity of OPLL, especially in patients with certain clinical and demographic characteristics in a Chinese population (Fig. 2 and Table S3). As mentioned above, we identified a 3 kb block of intense LD that included the translated region of exon 2 of the BMP9 gene and contained four SNPs. Among the four SNPs in this region, the additive models of rs75024165 (TT vs. CT vs. CC) and rs34379100 (CC vs. AC vs. AA) were associated with the occurrence of OPLL. rs34379100 is located in the 3′ untranslated region (3′-UTR) of BMP9. We suggest here for the first time that this synonymous SNP might affect the degree of post-transcriptional regulation in OPLL patients, with a resulting protein over expression. rs75024165 C>T is located in the translated region of BMP9 exon 2. This non synonymous SNP codes an amino acid change: the T allele forms part of a methionine codon, whereas the C allele is part of a codon for threonine what might affect the BMP9 protein function. Radiographic and histological evaluations have demonstrated that overproduction of BMP6 and BMP9 protein induces a more robust and larger ossification mass than other BMPs in an orthotopic ossification animal model [26]. Dumont and Helm found that direct or *ex vivo* BMP9 adenoviral gene therapy could induce massive bone formation at the injection sites and clearly promote spinal fusion on CT films [32], [33]. However, additional studies are needed to understand the functional effects of the CTCA haplotype *in vivo*. In addition the study on the effect of BMP9 polymorphism should be extended to the ossification of the thoracic and lumbar spine. We did not find OPLL patients in the CTCA haplotype who suffered from high FBG in the case group. Chen et al. indicated that human BMP9 proteins reduced glycemia to near-normal levels after a subcutaneous injection in diabetic mice. Overproduction of BMP9 might cause the same effect in regulating blood glucose concentration in OPLL patients with the haplotype CTCA [24], but a further analysis of this negative correlation is necessary.

We conclude that the BMP9 gene variants which are associated with susceptibility to OPLL in our Chinese population are located between rs9421799 and rs7923671. Among the four SNPs in this region, we obtained supporting evidence for an association between the occurrence of OPLL and rs75024165 and rs34379100. Moreover patients who carried the CTCA haplotype had a higher number of ossified vertebrae than those who did not (Fig. 2), but after analyzing the distribution of the number of ossified vertebrae in relation to clinical and demographic characteristics, smoking, regular drinking, normal FBG, middle age, male, regular sleeping habit, high BMD, normal exercise, and being over weighted were additional significant factors for the ossification severity in CTCA haplotype patients.

In summary, this study provides evidence for an association between a specific polymorphism in the BMP9 gene and the occurrence of OPLL. The CTCA haplotype is not only associated with the occurrence of OPLL but also with increased severity of OPLL in Chinese patients, when it does occur in combination with other clinical and demographic characteristics. The results of the current study may contribute to the understanding of the background mechanisms and molecular etiology of OPLL, which should lead to further biomedical or functional studies and recommendations for a healthy lifestyle that will reduce the incidence of this disease.

## Supporting Information

Table S1 **Primer pairs and their annealing temperatures, which have been used to amplify 5 BMP9 genomic areas spanning SNPs rs3758496, rs12252199, rs7923671, rs75024165, rs34379100 and rs9421799.**
(TIF)

Table S2 **Map of the** ***BMP9*** **gene region.** The upper bar on the left picture indicates exon 1 and the lower bar exon 2. The locations and rs-IDs of the 6 SNPs are given relative to the Ensembl ENSG00000128802 chromosome 10 sequence, P values of the alleles in our cohort and the amino acid substitutions for non-synonymous SNPs are indicated.
(TIF)

Table S3 **The distribution of ossified vertebrae in relation to the haplotype and clinical and demographic characteristics of the patients. Note:** The data are expressed as [number of CTCA carriers vs. non carriers in case group (mean of ossified cervical vertebrae±SD)].
(TIF)

Material S1 (DOC)
