# Supplementary figures and images for: Association of a BMP9 Haplotype with Ossification of the Posterior Longitudinal Ligament (OPLL) in a Chinese Population
Source: PLoS One. 2012 Jul 19;7(7):e40587. doi: 10.1371/journal.pone.0040587 (PMC3400650; doi:10.1371/journal.pone.0040587)

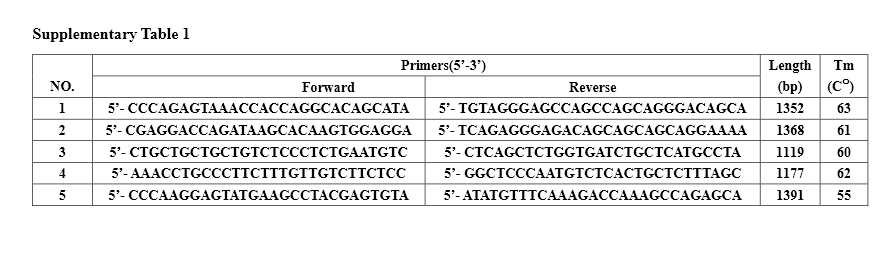

Supplement: Table S1 — Primer pairs and their annealing temperatures, which have been used to amplify 5 BMP9 genomic areas spanning SNPs rs3758496, rs12252199, rs7923671, rs75024165, rs34379100 and rs9421799. (TIF) [file pone.0040587.s001.tif]

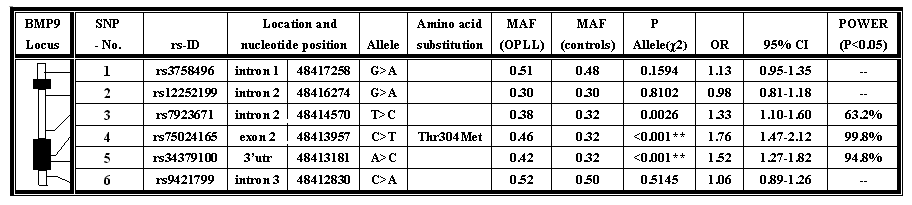

Supplement: Table S2 — Map of the BMP9 gene region. The upper bar on the left picture indicates exon 1 and the lower bar exon 2. The locations and rs-IDs of the 6 SNPs are given relative to the Ensembl ENSG00000128802 chromosome 10 sequence, P values of the alleles in our cohort and the amino acid substitutions for non-synonymous SNPs are indicated. (TIF) [file pone.0040587.s002.tif]

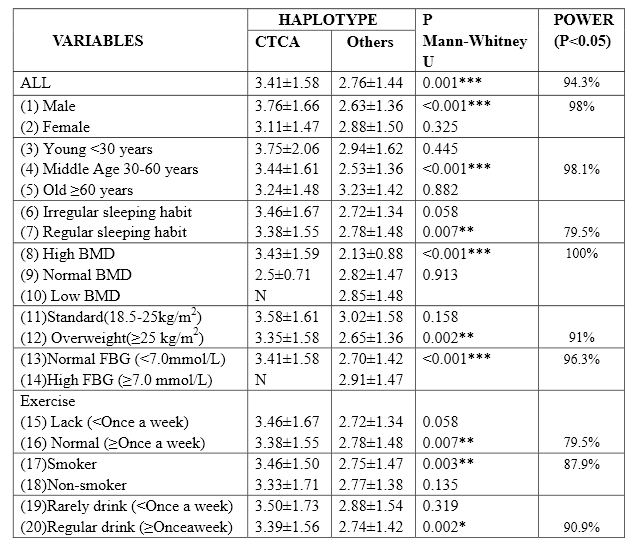

Supplement: Table S3 — The distribution of ossified vertebrae in relation to the haplotype and clinical and demographic characteristics of the patients. Note: The data are expressed as [number of CTCA carriers vs. non carriers in case group (mean of ossified cervical vertebrae±SD)]. (TIF) [file pone.0040587.s003.tif]
