# Supplementary material for: Association of a BMP9 Haplotype with Ossification of the Posterior Longitudinal Ligament (OPLL) in a Chinese Population
Source: PLoS One. 2012 Jul 19;7(7):e40587. doi: 10.1371/journal.pone.0040587 (PMC3400650; doi:10.1371/journal.pone.0040587)
Supplement: Material S1 — (DOC) [file pone.0040587.s004.doc]

**Supplementary Matrial S1**

**Standardized questionnaire**

The questionnaire included items on lifestyle factors: age (≥60 old, 30-60 middle age, <30 young, years), gender (male/female), regular sleeping habits (yes/no), physical exercise (≥once a week / <once a week), alcohol drinking (< once a week / ≥once a week), and smoking (yes/no).

**Anthropometric and Lifestyle Measurements**

The heights and weights of the subjects were recorded, and the body mass index (BMI) of all subjects was calculated using the weight/height^2^ formula. Overweight (BMI :> 25 kg/m^2^) or standard weight (BMI: 18.5-25 kg/m^2^) was defined according to the WHO criteria (1985). Fasting blood glucose (FBG) was measured with standard methods. High FBG (FBG concentration: ≥7.0mmol/liter (126mg/dl) was diagnosed according to the WHO criteria (1999). Bone mineral density (BMD g/cm^2^) of the distal radius in each subject was measured by dual-energy X-ray absorptiometry (DXA) using Lunar PIXI instrumentation (Madison, WI). A T-score was calculated using reference tables showing means and standard deviations. T-score cutoff values of <-1, -1-1, and ≥1 correspond to low, normal, and high BMD, respectively. All interviews were conducted by trained interviewers, and all results were evaluated using a standard protocol.
